# Optimized Solid-Phase Mesh-Enhanced Sorption from Headspace (SPMESH) for Rapid Sub-ng/kg Measurements of 3-Isobutyl-2-methoxypyrazine (IBMP) in Grapes

**DOI:** 10.3390/molecules27196195

**Published:** 2022-09-21

**Authors:** Terry L. Bates, Jessica Rafson, Hui Feng, Bruce S. Pan, Benjamin R. J. Mueller, Benjamin Yancey, William Fatigante, Gavin L. Sacks

**Affiliations:** 1 Department of Food Science, Stocking Hall, Cornell University, Ithaca, NY 14853, USA; 2 E&J Gallo Winery, Modesto, CA 95354, USA; 3 Bruker Daltonics, Billerica, MA 01821, USA

**Keywords:** DART-MS, SPMESH, SPME, volatile analysis, GC-MS, HRMS, methoxypyrazines

## Abstract

Parallel extraction of headspace volatiles from multiwell plates using sorbent sheets (HS-SPMESH) followed by direct analysis in real-time high-resolution mass spectrometry (DART-HRMS) can be used as a rapid alternative to solid-phase micro-extraction (SPME) gas-chromatography mass-spectrometry (GC-MS) for trace level volatile analyses. However, an earlier validation study of SPMESH-DART-MS using 3-isobutyl-2-methoxypyrazine (IBMP) in grape juice showed poor correlation between SPMESH-DART-MS and a gold standard SPME-GC-MS around the compound’s odor detection threshold (<10 ng/kg) in grape juice, and lacked sufficient sensitivity to detect IBMP at this concentration in grape homogenate. In this work, we report on the development and validation of an improved SPMESH extraction approach that lowers the limit of detection (LOD < 0.5 ng/kg), and regulates crosstalk between wells (<0.5%) over a calibration range of 0.5–100 ng/kg. The optimized SPMESH-DART-MS method was validated using Cabernet Sauvignon and Merlot grape samples harvested from commercial vineyards in the central valley of California (*n* = 302) and achieved good correlation and agreement with SPME-GC-MS (R^2^ = 0.84) over the native range of IBMP (<0.5–20 ng/kg). Coupling of SPMESH to a lower resolution triple quadrupole (QqQ)-MS via a new JumpShot-HTS DART source also achieved low ng/kg detection limits, and throughput was improved through positioning stage optimizations which reduced time spent on intra-well SPMESH areas.

## 1. Introduction

Several compounds in raw grapes have been linked to sensory attributes in wines, and thus may be useful for screening or sorting grapes based on intended wine quality [1,2,3]. One such odorant is 3-isobutyl-2-methoxypyrazine (IBMP, “herbaceous, bell pepper aroma”) [4]. IBMP accumulates primarily in the skins of grape berries before vèraison after which concentrations begin to decrease [4,5]. IBMP is absent in the mature fruit of many cultivars, but may be found above recognition threshold (15 ng/L in red wine; or, <15 ng/kg assuming a density of wine of 1 g/mL) and contribute to the varietal character of Bordeaux varieties (e.g., Cabernet Sauvignon, Sauvignon blanc, Cabernet franc, and Merlot) [4,5,6]. However, at excess concentrations, IBMP can diminish wine fruitiness, which may be detrimental to consumer acceptance [7].

As with other trace-level odorants in foodstuffs, the routine analysis of IBMP is complicated by its low concentration and the presence of many interfering compounds in the grape and wine matrix [8]. The extraction and preconcentration of IBMP and other odorants are often done by headspace solid phase microextraction (HS-SPME), as the approach requires no solvents and is readily automated [9,10]. After SPME extraction, odorants are thermally desorbed in the heated inlet of a gas chromatograph-mass spectrometer (GC-MS) for separation and detection. This approach is well-established, and low ng/L or ng/kg detection limits are feasible [11], but throughput is limited by both the HS-SPME extraction step (5 min or longer) and chromatography step (20–60 min) [8].

Solid-phase mesh-enhanced sorption from headspace (SPMESH) sheets coupled to direct analysis in real time mass spectrometry (DART-MS) has been reported as a rapid alternative to SPME-GC-MS for trace analysis of IBMP and other odorants [12,13]. SPMESH sheets are a variant of thin-film microextraction and consist of thin sorbent material (e.g., PDMS) etched to make it compatible with transmission mode DART-MS [14]. During use, the SPMESH sheet is positioned above a loaded multiwell plate for parallel headspace extraction of volatiles. After extraction, the SPMESH sheet is transferred to an automated positioning stage for DART-MS analysis of extracted spots. Using grape samples, this workflow was able to achieve ng/L detection limits for IBMP in 24 samples in 17 min [13]. However, run-to-run repeatability in grape homogenate was relatively poor (coefficient of variation (CV) = 32%). Additionally, headspace leakage among adjacent wells (‘crosstalk’) was high, approximately 1.2%, which limited the dynamic range of the approach [13]. Finally, while validation of SPMESH-DART-MS against HS-SPME-GCMS using commercial vineyard samples show acceptable correlation (R^2^ = 0.71) across an IBMP range of <2 to 28 ng/L (or <2 to 26 ng/kg, However, close inspection of the data reveals poor correlation between the methods when considered only those samples at or below the sensory threshold (<2 to 15 ng/L) [12,13].

The purpose of this study was (i) to develop an improved and fully optimized SPMESH-DART-MS/MS approach for quantitating IBMP at sub-ng/kg levels in grape homogenate by improving limit of detection and repeatability, (ii) to perform a large-scale validation study (*n* = 302) using grape samples from commercial vineyards, and (iii) to demonstrate the suitability of the approach on multiple MS detectors.

## 2. Results and Discussion

### 2.1. General Approach to Quantitating IBMP in Grape Homogenate Samples

**Figure 1:**
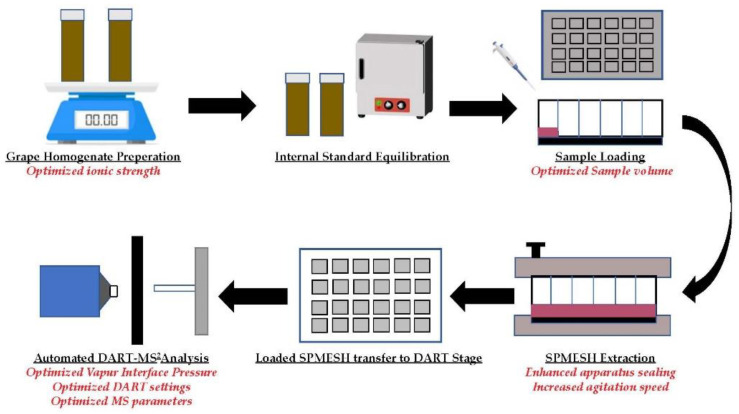
Overview of SPMESH-DART-MS/MS extraction and analysis approach including optimized sample preparation, SPMESH extraction, and DART-MS/MS parameters in this study.

This work describes improvements in sample preparation, extraction conditions, and DART-Orbitrap-MS (HRMS) settings for the analysis of sub-ng/kg levels of IBMP in grape homogenate. The optimized SPMESH-DART-MS workflow was validated against the gold-standard SPME-GC-MS method using vineyard samples with sub-odor threshold concentrations of IBMP. The methodology was also evaluated on a lower cost triple quadrupole MS detector (QqQ-MS). Finally, the use of a new DART design (JumpShot-HTS) that facilitates better control of the X-Y positioning stage was also evaluated. The SPMESH-DART-MS workflow has been described in previous studies, and consists of the following steps (Figure 1):

- Grape samples are homogenized, combined with saturated NaCl, and spiked with isotopically labelled internal standard.
- Prepared samples are placed in a heated agitator for internal standard equilibration.
- Samples are transferred to a 24-well plate, placed in the SPMESH extraction apparatus and incubated in a heated agitator for SPMESH extraction.
- The loaded SPMESH is removed from extraction apparatus and transferred to the automated X-Y positioning stage of an SVP-DART or JumpShot-DART ion source coupled to an appropriate MS and analyzed.

### 2.2. Optimization of Sample Preparation and Extraction Conditions

In previous work using SPMESH-DART-HRMS/MS for rapid analyses of IBMP reported repeatability (RSD (relative standard deviation) = 32%) and limits of detection (LOD = 4 ng/L, or 3.6 ng/L assuming a homogenate density of 1.1 g/mL) when quantifying native IBMP in grape homogenate [13], worse than what can be achieved with the gold standard HS-SPME-GCMS approach (RSD = 2–14%, LOD = 1.2 ng/L or ng/kg in wines) [11]. During SPMESH sheet extraction, the sheet is placed in a stainless-steel holder, and the wells are isolated from each other via a Teflon gasket containing water jet cut holes to expose the mesh. The stainless-steel sheet holder and gaskets are placed above the well plate prior to sealing the extraction apparatus with bolts at the corners of the apparatus. The original SPMESH sheet holder allowed for modest levels of leakage between adjacent wells and averaged 1.2% per well [13]. For central wells, which are adjacent to four other wells, average crosstalk is 4 × 1.2% = 4.8%, which limits the dynamic range of samples that could be analyzed on a single plate. This original SPMESH holder design was used for a previous validation study with IBMP in grape samples [13]. Recently, a new SPMESH holder design was described to allow for ‘direct-immersion’ SPMESH in which the sample holder can be inverted without leakage [15]. Briefly, the new extraction apparatus adds a second solid Teflon gasket on top of the SPMESH holder and below the metal cover plate. Additionally, a vise clamp is used in place of butterfly nuts to ensure that uniform pressure is applied across the central and peripheral well plates. The new SPMESH holder design limited cross talk to <0.03% per well for IBMP analyses, but other analytical figures of merit were not reported.

**Figure 2:**
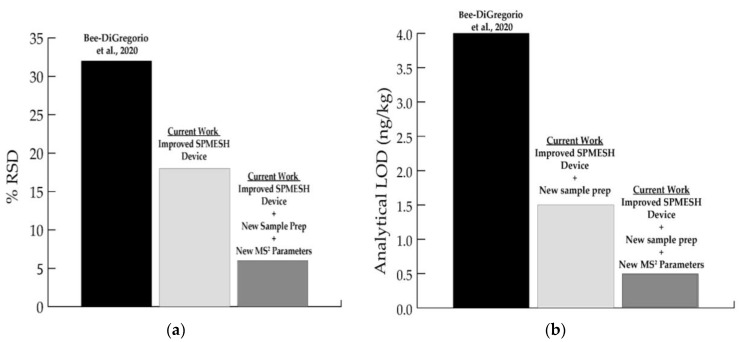
Comparison of reported relative standard deviation values from previous work (Bee et al., 2020) [13], samples analyzed with improved SPMESH extraction device, and samples analyzed using improved SPMESH extraction device and optimized conditions (**a**). Comparison of analytical limits of detection from (i) Bee-DiGregorio et al., 2020 [13], (ii) current work with optimized sample prep and new device, and (iii) improved device, optimized sample prep, and optimized MS/MS parameters (**b**).

Using IBMP spiked at 5 ng/kg into grape homogenate (*n* = 8), the repeatability of measurements was determined to be 18% RSD. This result is a considerable improvement over the repeatability reported (32% RSD, Figure 2) using the older SPMESH holder design but otherwise identical conditions [12]. Further improvements in limit of detection and repeatability were achieved by increasing the mass of grape homogenate loaded into each well from 3.5 to 5 g, which resulted in a 41% increase in signal at 5 ng/kg (*n* = 8, *p* < 0.05). Because the increase in signal was roughly the same as the increase in sample size, a likely explanation is that the increased sample mass led to a proportional increase in the mass of IBMP extracted (10 pg vs. 14 pg) [9]. This sample-size-dependent signal is more commonly observed with thin-film geometries such as SPMESH as compared to SPME fibers due to the higher sorbent volume of the former [16,17]. This change, in combination with apparatus and sample preparation optimization, led to a decrease in the limit of detection by more than half 3.6 ng/kg vs. 1.5 ng/kg).

### 2.3. Optimization of DART-HRMS/MS Conditions

DART desorption temperatures were evaluated over a range from 250 to 450 °C. Testing was performed using SPMESH sheets following extraction of IBMP spiked into saturated NaCl solution at 40 ng/L. The optimal temperature for IBMP response was at 350 °C showed a 10-fold signal increase as compared to the previous setting of 450 °C (data not shown). The lower response at temperatures below 350 °C was likely due to incomplete thermo-desorption of the analyte from the SPMESH sheet. The decreased response at higher temperatures was likely due to increased fragmentation of the precursor ion reducing the amount of the target mass reaching the MS detector, as has been reported for other DART-MS analyses, e.g., of n-hexadecane [18]. The pressure setting for the VAPUR Interface pump was also evaluated, with maximum signal intensity (22% increase at 5 ng/kg as compared to previous work) observed at a setting of (9.5 × 10^4^ Pa). In previous work on SPMESH-DART-MS when the pump was set to the highest setting (8.8 × 10^4^ Pa), a significant number of ions were evacuated from the VAPUR Interface without entering the vacuum entrance chamber of the mass spectrometer [19]. Conversely, at a low setting, less ions are sampled from the reaction zone, limiting signal intensity.

Mass transitions, collision energies, and analytical cycle time were optimized on the Orbitrap-MS to further improve sensitivity and selectivity [20]. A new MS-MS transition was identified for d_3_-IBMP and collision energies were optimized (25 eV to 17 eV) for both IBMP and d_3_-IBMP. An isobaric interference was noted at the previous transition (*m/z* 170 ➔ *m/z* 127.0818) which was resolved at the new transition (*m/z* 170.1351 ➔ *m/z* 128.0896). Scan density—a critical parameter for sensitivity [20,21]—was increased by reducing mass resolution to from 140 K to 70 K, reducing automatic gain control (AGC) from 10^6^ to 10^5^, and reducing injection time from 250 ms to 100 ms. These changes more than doubled the sampling rate. In combination, the improvements to the DART-MS parameters led to increased repeatability (5% RSD) and significant decreases in the limit of detection (<0.5 ng/kg) (Figure 2).

### 2.4. Method Calibration and Figures of Merit

**Figure 3:**
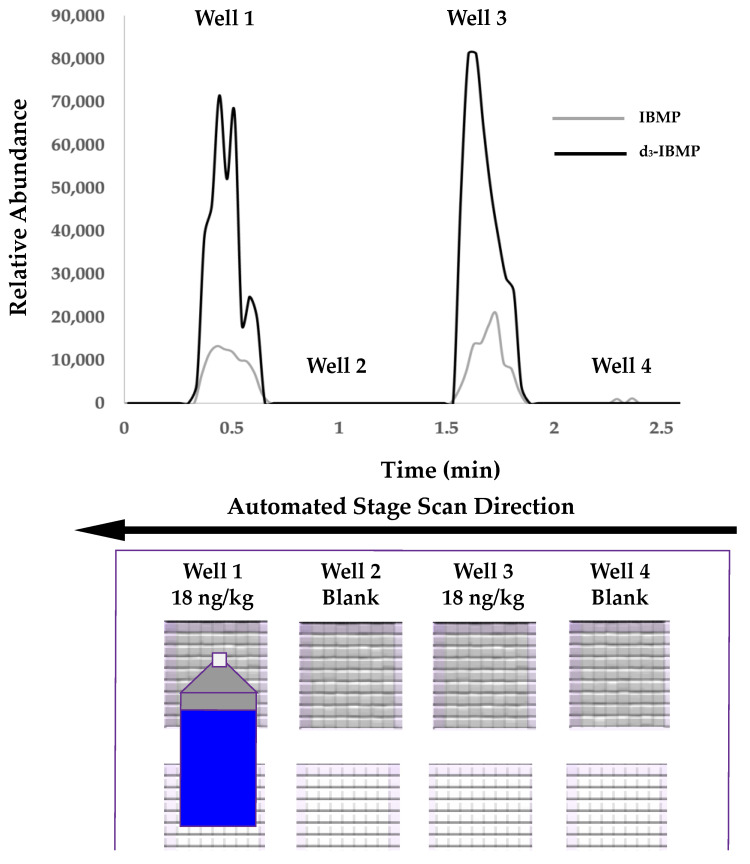
DART-Orbitrap-MS/MS selected ion chronograms for IBMP (*m/z* 167.1427 ➔ *m/z* 124.0632) at 18 ng/kg and d_3_-IBMP at 100 ng/kg (*m/z* 170.1351 ➔ *m/z* 128.0896) in *Vitis vinifera* (cv. Barbera) grape homogenate with no detectable IBMP as previously determined by HS-SPME-GCMS. Wells 1 and 3 are analytical replicates spiked with IBMP at 18 ng/kg and d_3_-IBMP at 100 ng/kg and wells 2 and 4 are blank wells.

Calibration standards of IBMP were prepared at 6 levels (0.5–100 ng/kg) in a *Vitis vinifera* (cv. Barbera) homogenate with no detectable concentration of IBMP as previously determined by HS-SPME-GC-MS. An isotopically standard (d_3_-IBMP) was added at 100 ng/kg to all standards. The use of isotopically matched internal standards to increase precision and accuracy in SPME extraction approaches, including SPMESH extraction, is well documented in the literature [22,23,24]. A representative HRMS selected ion chronogram (SIC) for IBMP and d_3_-IBMP at 18 ng/kg and 100 ng/kg, respectively, in Barbera grape homogenate is show in Figure 3.

Linearity, limits of detection, recovery, and repeatability for IBMP analyses using SPMESH-DART-HRMS/MS were compared with SPME-GC-MS for similar calibration samples, and to previous SPMESH-DART-HRMS/MS work in Table 1. The optimized SPMESH-DART-HRMS/MS approach in the current work yielded an LOD of <0.5 ng/kg as compared to 4 ng/kg in a previous study [13]. Improvements in precision were reduced from 32% RSD in previous work to 5% RSD in our current approach. Recovery for DART-HRMS/MS at 5 ng/kg was acceptable (110%). The figures of merit for SPMESH-DART-HRMS/MS were also comparable to those achieved with the standard SPME-GC-MS approach (Table 1). Improvements in the limit of detection and repeatability are a result of improvements in extraction apparatus, sample preparation, DART source parameters, and MS/MS parameters.

### 2.5. Validation of SPMESH-DART-MS/MS against HS-SPME-GC-MS

**Figure 4:**
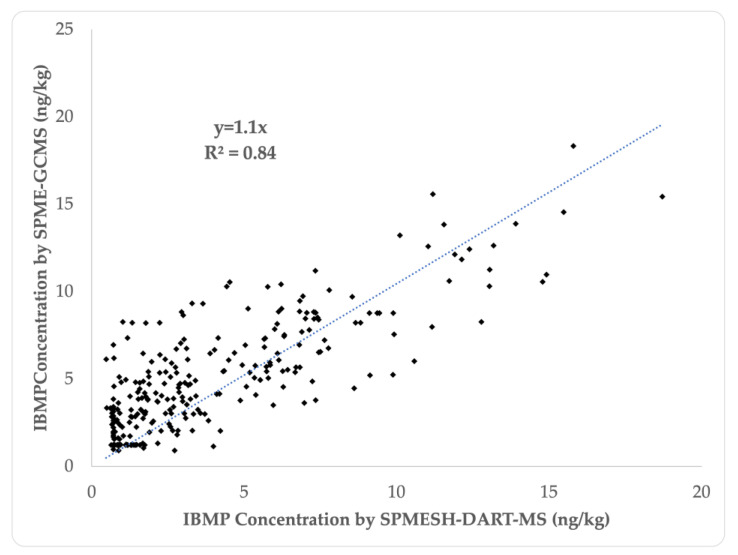
Correlation between IBMP measured by SPMESH-DART-Orbitrap MS/MS and HS-SPME-GC-MS. Undetectable values were set to the square root of the LOD (SPME-GC-MS = 1.2 ng/kg, SPMESH-DART-MS = 0.71 ng/kg).

For validation, post-veraison Cabernet Sauvignon and Merlot grapes were collected from multiple commercial vineyard sites in California (*n* = 302 samples). IBMP was quantified by SPMESH-DART-HRMS/MS using the newly optimized protocol, and by the gold standard HS-SPME-GC-MS methodology. Using, SPMESH-DART-HRMS/MS, concentrations of IBMP ranged from undetectable (<0.71 ng/kg) to 17 ng/kg, with a mean value of 3.9 ± 0.4. This range is representative of values reported elsewhere for these cultivars (8–15 ng/L) [10]. The newly optimized SPMESH-DART-HRMS/MS method was well correlated with SPME-GC-MS (R^2^ = 0.84, *p* < 0.05) with a slope of near unity at 1.1 (Figure 4). The root mean squared error between the two methods was 1.28 ng/kg.

**Figure 5:**
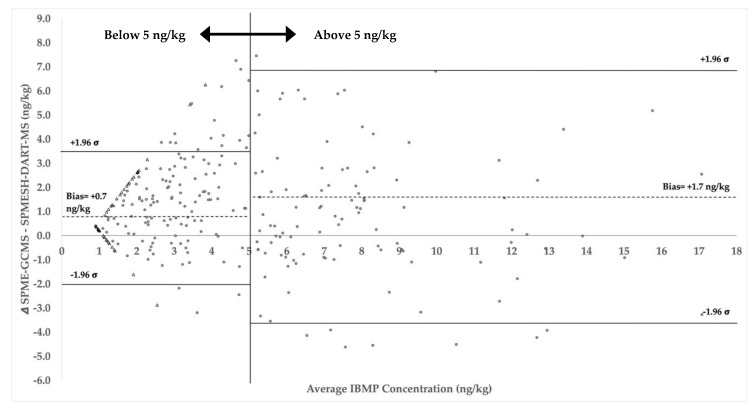
Bland–Altman agreement analysis for native IBMP measured in Merlot and Cabernet Sauvignon grapes harvested at commercial maturity in 2021 (*n* = 302). Bias (dotted lines) and upper/lower limits of agreement (solid lines) are shown represent average concentrations above and below 5 ng/kg. Samples for which IBMP was undetectable by one or both methods are shown as hollow triangles, and samples for which IBMP was detectable using both methods are shown as closed circles.

Performance of the SPMESH-DART-HRMS/MS method against HS-SPME-GCMS was further assessed by Bland–Altman analysis, in which the difference between analytical methods for each sample is plotted as a function of sample concentration (Figure 5) [25,26]. Separate calculations were performed for concentrations above and below 5 ng/kg because there was a noticeably higher variance at concentrations above this value. For concentrations below 5 ng/kg, the 95% confidence limit was ±2.6 ng/kg, with negligible bias from the origin (0.7 ng/kg). For concentrations in the range of 5–17 ng/kg, the 95% confidence limit was ±5.5 ng/kg, again with a negligible bias (1.7 ng/kg). Concentrations of IBMP in red wines are typically about 67% of their concentrations in fruit, and the sensory threshold of IBMP in red wines has been reported to be 15 ng/L (~15 ng/kg) [6,27]. Thus, the new SPMESH-DART-HRMS/MS method should be a reliable alternative to HS-SPME-GCMS for identifying fruit likely to have suprathreshold concentrations of IBMP.

### 2.6. Comparison of JumpShot-HTS DART-QqQ-MS/MS and SVP-DART-SPMESH-Orbitrap-MS/MS

**Figure 6:**
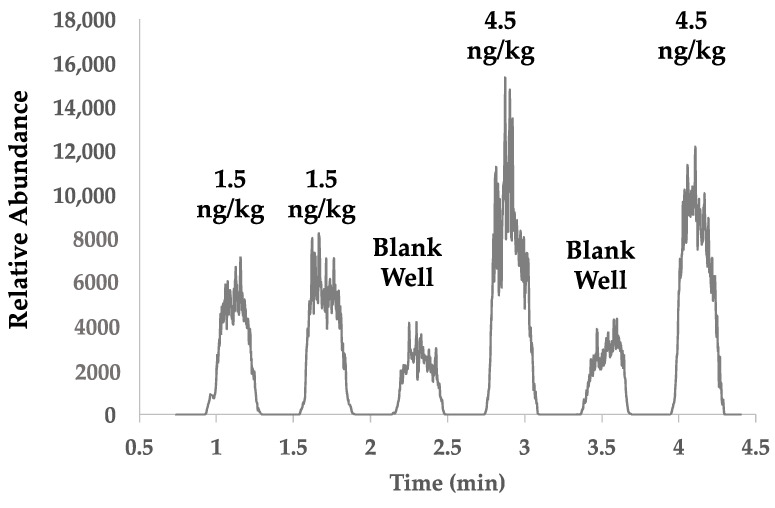
DART-QqQ-MS/MS selected ion chronograms for IBMP (*m/z* 167.4 ➔ *m/z* 124.1) at 1.5 ng/kg in *Vitis vinifera* (cv. Barbera) grape homogenate with no detectable IBMP as previously determined by HS-SPME-GCMS. Wells 1 and 2 are analytical replicates spiked with IBMP at 1.5 ng/kg, wells 4 and 6 are spiked with IBMP at 4.5 ng/kg, and wells 3 and 5 are blank wells.

Since HRMS detectors are less commonly associated with routine, quantitative analyses, the ruggedness of the optimized SPMESH-DART-MS method was assessed using a DART ion source coupled to a QqQ-MS/MS detector. Switching to a chromatography-free QqQ-MS/MS approach risked unacceptable limits of detection due to isobaric interferences often associated with ambient ionization based mass spectrometry [28]. The MS/MS transitions and collision energies were optimized for the QqQ-MS/MS analysis (*m/z* 167.4 ➔ *m/z* 124.6, CE = 20 eV), while all other parameters for the SPMESH extraction and DART ionization remained unchanged. Figures of merit are summarized in Table 1. As expected, the DART ionization coupled to a lower resolution instrument had background noise (~0.5 ng/kg; Figure 6). SPMESH-HRMS/MS demonstrated a higher LOD (<0.5 ng/kg vs. 1.5 ng/kg) due to absence of isobaric interferences. Method calibration was successfully performed without the use of an internal standard, and the average repeatability was adequate (5% RSD). However, repeatability on the HRMS without internal standardization was poor (17% RSD). We expect repeatability and LOD of the SPMESH-DART-QqQ-MS/MS approach to be enhanced with the addition of the deuterated internal standard [29].

During QqQ-MS/MS analyses, a JumpShot-HTS DART source was used in place of the SVP-DART ion source. The X-Y positioning stage of the JumpShot includes a second linear rail, which allowed for the automated movement of the ion source from row to row. The JumpShot-HTS also allows users to use variable scan rates or jump from location to location, as opposed to the constant scan speed required by the SVP-DART. Currently, approximately half of a SPMESH-DART-MS/MS analysis is spent collecting data from the inter-well regions of the sheet that contain no extracted volatiles (see Figure 6). We were able to program the JumpShot-HTS to move rapidly between spots of the SPMESH sheet (0.5 mm/s scan rate for spots, 10 mm/s between wells) which decreases total run time for a 24 well plate to ~8.5 min (from 17 min).

### 2.7. Conclusions

An optimized, rapid thin-film microextraction protocol using sorbent sheets (SPMESH) followed by DART-MS for trace level analyses of a key odorant (IBMP) in grape macerates below its sensory threshold was described. The improved method utilized a new SPMESH device holder design to prevent well-to-well leakage, increased sample size, and optimized DART and HRMS/MS settings. The new method achieved excellent agreement with a gold standard approach (SPME-GC-MS) at IBMP concentrations < 5 ng/kg, below the sensory threshold of IBMP. Low detection limits, <2 ng/kg, could also be achieved with a lower cost DART-QqQ-MS/MS instrument. Control of the X-Y positioning stage with JumpShot-HTS improves throughput by allowing for automated movement from row to row, and to sample only from regions containing extracted volatiles. The improvements described here for SPMESH-DART-MS should be expandable to rapid analyses of other trace level volatiles.

## 3. Materials and Methods

### 3.1. Materials, Chemical Reagents and Standards

Square well, polypropylene, 10 mL, 24-well plates were purchased from Analytical Sales and Services (Flanders, NJ, USA). PDMS sheets (CultureWell Silicone Sheet, 18 cm × 13 cm × 0.25 mm) were purchased from Grace Biolaboratories (Bend, OR, USA). PDMS sheet laser etching was performed at Cornell NanoScale Science and Technology Facility (Ithaca, NY, USA). Teflon gaskets were water jet cut and stainless-steel holders for SPMESH extraction apparatus were machined at the Cornell University LASSP machine shop (Ithaca, NY, USA). Twenty mL amber vials and PFTE lined screw caps were purchased from Supelco (Bellfonte, PA, USA). Sodium chloride and HPLC grade methanol were purchased from VWR (Radnor, PA, USA). 2-methoxy-3-isobutylpyrazine (IBMP) (>99% purity) was purchased from Sigma-Aldrich (St. Louis, MO, USA) and d_3_-IBMP (>98% purity, 99% D) was purchased from C/D/N Isotopes (Pointe-Claire, Quebec, CA, USA). Grapes used for calibration (*Vitis vinifera* cv. Barbera) were harvested at commercial maturity by E&J Gallo Winery (Modesto, CA, USA) from a single vineyard (California, Central Valley AVA) in September of 2021. Grapes used for validation (*Vitis vinifera* cv. Cabernet Sauvignon and cv. Merlot) were harvested at commercial maturity between July and October of the 2021 harvest by E&J Gallo Winery (Modesto, CA, USA) from multiple commercial California vineyards (Central Vineyard AVA).

### 3.2. Grape Sample Preparation: Optimization of Conditions

De-stemmed grapes were homogenized in a blender (Vitamix, Olmsted Falls, OH, USA) and stored at −20 °C until ready for use. To evaluate the effects of sample preparation techniques on SPMESH-DART-MS analysis, pre-frozen Barbera homogenate with undetectable native IBMP was used, and spiked with IBMP (5 ng/kg). Initially, 10.5 g of grape homogenate and 4.5 g saturated NaCl was transferred to a 20 mL amber vial and spiked with 10 µL of an internal standard solution (100 ng/kg). Vials were equilibrated at 70 °C and agitated at 230 RPM for 1 h in a Innova 4080 Incubator (New Brunswick Scientific, Edison, NJ, USA). 5 g aliquots (*n* = 8) were transferred to into wells of 24 well plate prior to SPMESH extraction. After optimization, 12 g of grape homogenate and 6 g saturated NaCl were transferred to a 20 mL vial and spiked with an internal standard solution (12.5 ng/kg). Vials were equilibrated at 70 °C and agitated at 300 RPM for 40 min. Finally, 7.5 g aliquots (*n* = 8) were transferred into wells of a 24-well plate prior to SPMESH extraction.

### 3.3. SPMESH Extraction Conditions

The general approach to SPMESH-DART-MS is depicted in Figure 1 and was adapted from previous work [12]. SPMESH Sheet and Teflon gaskets were baked out at 250 °C for 20 min in a laboratory convection oven immediately prior to extraction. Processed samples or calibration standards were loaded onto a 24-well plate and secured in the stainless-steel extraction apparatus. Next, a Teflon gasket followed by the SPMESH sheet in a stainless-steel holder, a solid Teflon gasket, and a stainless-steel cover were placed over the loaded plate. The extraction apparatus was tightly clamped and then secured in an incubator and agitated (250 RPM) at 55 °C for 45 min.

The SPMESH sheet in the stainless-steel holder was removed from the extraction apparatus and transferred to the X-Y positioning stage of a standardized voltage and pressure (SVP) DART ion source (IonSense Inc., Saugus, MA, USA). The DART ion source was coupled to either a Q Exactive mass spectrometer (Thermo Fisher Scientific, Waltham, MA, USA) through a VAPUR Interface with a pump setting of 9.5 × 10^4^ Pa (IonSense Inc., Saugus, MA, USA)**.** The VAPUR Interface was fitted with a vacuum pump to evacuate excess discharge gas prior to entering the MS inlet (Welch-IlmVac; Gardner Denver, Inc., Niles, IL, USA). For SPMESH-DART-HRMS/MS analysis, web-based DART software (version 6.0.5, IonSense Inc., Saugus, MA, USA) was used to manually move the SPMESH sheet on the DART positioning stage unidirectionally through the DART reaction zone at 0.5 mm/sec at a desorption temperature of 350 °C in front of the MS inlet. The DART ion source, fitted with a 2.5 mm ID insulator cap, was operated in positive ion mode with a grid voltage of 350 V using ultra high purity He as the carrier gas at 5.2 × 10^5^ Pa.

### 3.4. Orbitrap SPMESH-DART-HRMS/MS: Optimization of Conditions and Determining Figures of Merit

Optimization experiments were performed using spiked grape macerate for SPMESH-DART-MS analyses. To optimize scan density, Q Exactive MS analytical cycle time was varied by changing the maximum injection time from 250 ms to 100 ms in 50 ms increments. The effect of automatic gain control (AGC) was evaluated at 10^6^ and 10^5^. The effect of scan resolution was evaluated at 70,000 and 140,000. After optimization, the MS was operated in positive ion mode at a scan resolution of 70,000, 1 microscan, 100 s maximum injection time, automatic gain control (AGC) of 10^5^ over a mass range of *m/z* 75–180. IBMP and d_3_-IBMP were measured by MS/MS in parallel reaction monitoring mode (PRM) with mass tolerance of 5 ppm. The MS/MS transitions were identified in previous work [12]. Precursor ion for d_3_-IBMP was identified by dropping 10 µL of standard solutions in the DART reaction zone while operating the MS in full scan mode over mass range of *m/z* 75–180. Product ion scans were performed in a similar manner; however, the MS was operated in PRM mode. Transitions monitored were: *m/z* 167.1427→124.0632 at CE 17 eV for IBMP and *m/z* 170.1351→128.0896 at CE 17 eV for d_3_-IBMP. For method calibration and determining linearity, repeatability, and limits of detection, six calibration standards (range = 0.5–100 ng/kg IBMP; 100 ng/kg d_3_-IBMP) were prepared in Barbera grape macerate, as described above, and analyzed in replicate. To determine recovery, samples were spiked with 5 ng/kg IBMP. LOD and LOQ were calculated using calibration data based on S:N = 3 and S:N = 10, respectively.

### 3.5. Analyisis of IBMP in Grape Macerate by HS-SPME-GC-MS: Conditions and Figures of Merit

Quantification of IBMP in validation samples was carried out by headspace solid phase microextraction (HS-SPME; Gerstel Multipurpose Sampler Gerstel Inc., Linthicum, MD, USA) coupled to an Agilent 7890B-5977 MSD (Agilent, Santa Clara, CA, USA) GC-MS system using a method similar to that described by Bee-DiGregorio et al., 2020 [13]. Grape macerate samples were prepared as described for SPMESH samples, except that a 7 g sample was combined with 10 µL internal standard solution, 20 µL sodium dodecylsulfate (SDS) to disrupt enzymatic activity and 3 mL saturated NaCl solution to increase volatility before loading into 20 mL amber autosampler vials. HS-SPME analyses were performed using a 1 cm 50/30 µm divinylbenzene-Carboxen-polydimethylsiloxane (DVB/CARB/PDMS; Supelco, Bellafonte, PA, USA) was pre-incubated temperature of 70 °C for 1 min followed by an 18 min HS-SPME extraction at 70 °C. The SPME fiber was desorbed for 300 s in a multimode injector at 250 °C in splitless mode for 2 min with a purge time of 0.85 min, a purge flow of 50 mL/min and a septum purge flow of 3 mL/min. The GC column was a DB-WAX UI column (Agilent, Santa Clara, CA, USA) (30 m × 0.25 mm × 0.50 µm) with ultra-high purity helium as a carrier gas at a flow rate of 1.0 mL/min. The GC oven was held at 42 °C for 1.5 min then ramped to 75 °C at 11 °C/min and held for 0.2 min, increased to 135 °C at 6 °C/min and held for 0.2 min, increased to 150 °C at 11 °C/min, and then increased to 240 °C at 120 °C/min and held for 2 min for a total run time of 19.0 min. The MS was operated in EI mode at an ionization energy of 70 eV with an ion source temperature of 230 °C, a quadrupole temperature of 150 °C and interface temperature of 240 °C. For calibration and determining figures of merit, frozen and thawed Barbera grape macerate was spiked with varying levels of IBMP (6.25, 12.5, 25, 50, 100, 200, 400 ng/kg) in replicate. Recovery experiments in grape macerate were performed in replicate at 5 ng/kg, as described for SPMESH-DART-HRMS/MS.

### 3.6. JumpShot-HTS-DART-QqQ-MS/MS Conditions

For SPMESH-DART-QqQ-MS/MS, a JumpShot-DART with a high throughput screening (HTS) package (IonSense; Saugus, MA, USA) was coupled to a Bruker EVOQ Elite QqQ MS (Bruker Daltronics, Billerica, MA, USA). During analyses of IBMP extracted onto SPMESH sheets, the JumpShot-HTS X-Y positioning stage scanned across a row a constant rate of 0.5 mm/s, and moved from row to row automatically. A program was also written to allow the X-Y positioning stage to scan at 0.5 mm/s over extracted spots, and at 10 mm/s over inter-well regions. The MS was operated in positive ion mode with a scan speed of 100 ms in multiple reaction monitoring (MRM) mode. The collision energy (CE = 20 eV) for the *m/z* 167.4 ➔ *m/z* 124.6 transition was optimized by dropping 10 µL standard solutions of IBMP in the DART reaction zone while operating the MS in MRM mode.

## Figures and Tables

**Table 1:** Figures of merit for SPMESH-DART-MS/MS of IBMP in grape homogenate.

	Bee-DiGregorio, 2020 [13]	Current Work	Current Work	Current Work
	SPMESH-SVP-DART-HRMS/MS	HS-SPME-GCMS	SPMESH-SVP-DART-HRMS/MS	SPMESH-JumpShot HTS-DART-QqQ-MS/MS
**Calibration Range**	6.25–400 ng/L	3.4–540 ng/kg	0.5–100 ng/kg	1.5–40 ng/kg
**R^2^**	0.99	0.99	0.99	0.97
**LOD**	4 ng/L (3.6 ng/kg) ^a^	1.2 ng/kg	<0.5 ng/kg	1.5 ng/kg
**LOQ**	12 ng/L (10.9 ng/kg) ^a^	4 ng/kg	1.5 ng/kg	5 ng/kg
**Recovery (@ 5 ng/kg)**	n.d.	93%	110%	n.d.
**Average %RSD**	32%	6%	5%	5%
**Throughput (total MS analysis time for 24 samples)**	17 min	17.3 h	17 min	8.5 min ^b^

^a^ Assuming density of grape homogenate to be 1.1 g/mL. ^b^ Throughput achievable by programming X-Y positioning stage to rapidly scan (10 mm/s) through inter-well regions of SPMESH sheet which lack extracted volatiles. Otherwise, if constant 0.5 mm/s scan rate is used for all regions, throughput is 17 min for 24 samples. n.d. = not determined

## Data Availability

Raw data can be made available upon request to the corresponding author.
